# Supplementary material for: Ovulation-Derived Fibronectin Promotes Peritoneal Seeding of High-Grade Serous Carcinoma Precursor Cells via Integrin β1 Signaling
Source: Cells. 2026 Jan 4;15(1):80. doi: 10.3390/cells15010080 (PMC12785380; doi:10.3390/cells15010080)
Supplement: Supplementary file 1 [file cells-15-00080-s001.zip › cells-3960409-supplementary.pdf]

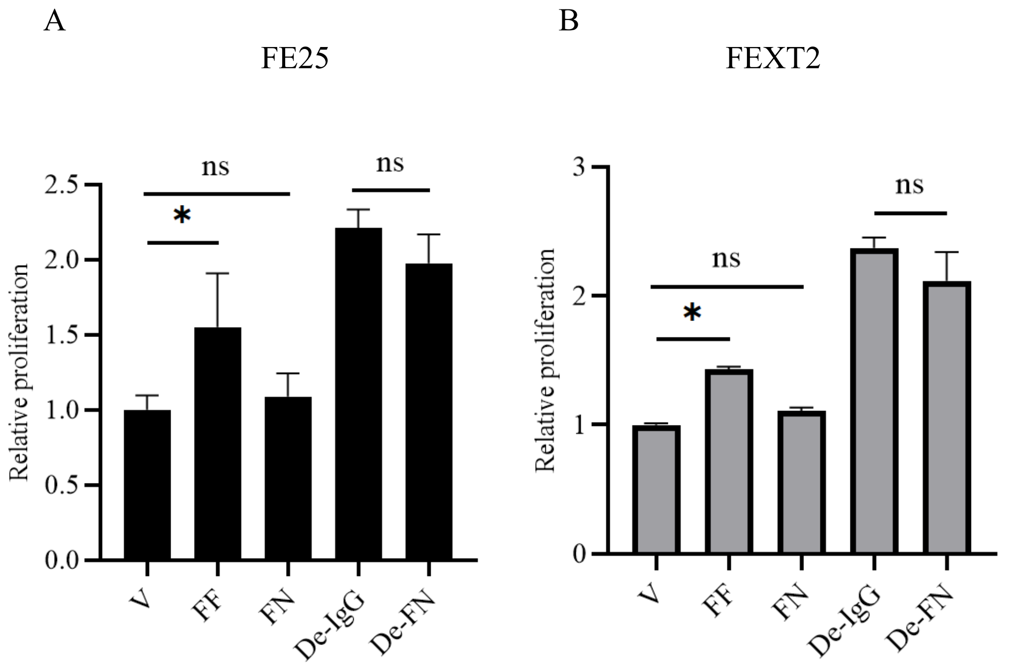

Figure S1: Proliferation analysis of FTE cells treated with FF, FN, and FN-depleted FF (De-FN)

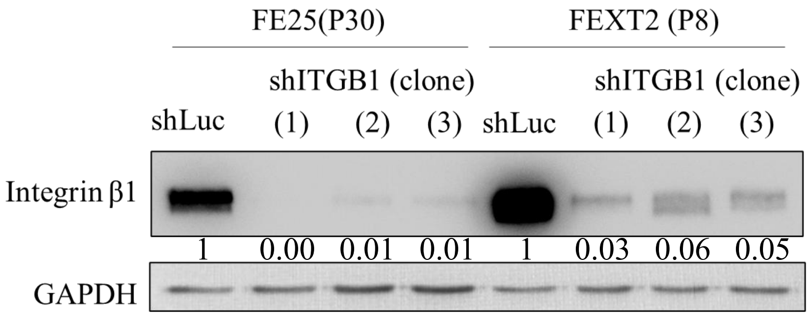

Figure S2:Western blot analysis of ITGB1 knockdown in FTE cells

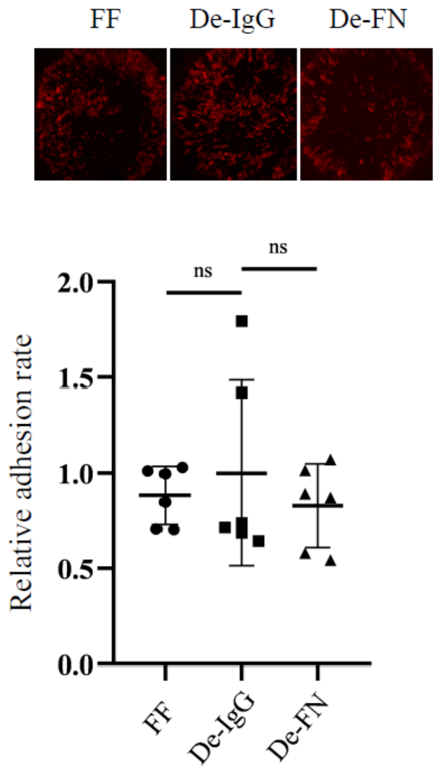

Figure S3: Ex vivo peritoneal adhesion analysis of FEXT2 cells treated with FF, De-IgG, and De-FN.

Supplementary Table S1: Antibody details

Antibody		Source (catalogue No.)	Dliution
Primary	p-AKT	Cell signaling (#4060)	WB 1:1000
	p-SRC	Cell signaling (#2101)	WB 1:1000
	p-FAK	Cell signaling (#3283)	WB 1:1000
	FN	Genetex (GTX112794)	WB 1:1000
	ITGB1	Genetex (GTX128839)	WB 1:1000
	GAPDH	Santa Cruz (sc-32233)	WB 1:1000
Secondary	Goat anti-rabbit IgG-HRP	Santa Cruz (sc-2030)	WB 1:5000
	Goat mouse IgG-HRP	Santa Cruz (sc-2005)	WB 1:5000
